# Evaluating the Influence of Epidemiological Parameters and Host Ecology on the Spread of Phocine Distemper Virus through Populations of Harbour Seals

**DOI:** 10.1371/journal.pone.0002710

**Published:** 2008-07-16

**Authors:** Catriona M. Harris, Justin M. J. Travis, John Harwood

**Affiliations:** 1 Sea Mammal Research Unit, University of St. Andrews, St Andrews, Fife, United Kingdom; 2 Centre for Research into Ecological and Environmental Modelling, University of St Andrews, St Andrews, Fife, United Kingdom; 3 School of Biological Sciences, University of Aberdeen, Aberdeen, Aberdeenshire, United Kingdom; University of Liverpool, United Kingdom

## Abstract

**Background:**

Outbreaks of phocine distemper virus (PDV) in Europe during 1988 and 2002 were responsible for the death of around 23,000 and 30,000 harbour seals, respectively. These epidemics, particularly the one in 2002, provided an unusual opportunity to estimate epidemic parameters for a wildlife disease. There were marked regional differences in the values of some parameters both within and between epidemics.

**Methodology and Principal Findings:**

We used an individual-based model of seal movement that allowed us to incorporate realistic representations of space, time and animal behaviour into a traditional epidemiological modelling framework. We explored the potential influence of a range of ecological (foraging trip duration, time of epidemic onset, population size) and epidemiological (length of infectious period, contact rate between infectious and susceptible individuals, case mortality) parameters on four readily-measurable epidemic characteristics (number of dead individuals, duration of epidemic, peak mortality date and prevalence) and on the probability that an epidemic would occur in a particular region. We analysed the outputs as if they were the results of a series of virtual experiments, using Generalised Linear Modelling. All six variables had a significant effect on the probability that an epidemic would be recognised as an unusual mortality event by human observers.

**Conclusions:**

Regional and temporal variation in contact rate was the most likely cause of the observed differences between the two epidemics. This variation could be a consequence of differences in the way individuals divide their time between land and sea at different times of the year.

## Introduction

Phocine distemper virus (PDV) was first identified in 1988 when it killed more than 23,000 harbour seals around Europe [1]–[4]. It swept through European seal populations over a period of 9 months and was not observed again until May 2002, when unusual levels of mortality attributed to PDV [5], [6] were reported from the same location (the island of Anholt in Denmark) where the 1988 epidemic started. An estimated 30,000 harbour seals died in the 2002 epidemic, again within a 9 month period [5].

In both epidemics, intensive effort was made to ensure that the numbers of dead animals washed ashore were recorded, together with additional information on their species, sex and age [4], [7], [8]. These data have been used to estimate basic epidemiological parameters for both years on a regional basis. The number of seals that died was estimated both from counts of carcasses and from surveys carried out before and after the epidemics. In addition, estimates of the peak mortality date (PMD = the date at which 50% of the final asymptotic mortality was reached) and the duration of epidemic (DOE = the number of days during which 90% of carcasses were found, with the mean fixed at the PMD) were available for most regions [2], [8], [9]. In the UK, the prevalence of antibodies in the surviving populations was also determined after both epidemics (results summarised in [10], [11]). These data can be used to determine the number of individuals that came into contact with the disease, the contact rate (the number of susceptibles that an infective individual would infect in a naïve population during its infectious period) and the case mortality (the probability of an infected individual dying) for each local population [11].

In both 1988 and 2002 the epidemic started early in the year, and then spread throughout all European harbour seal populations. It reached the UK towards the end of both years, and faded out after reaching Scotland [8]. In both years, mortality rates decreased over the duration of the epidemic. For example, in 1988 the mortality rates had declined from >50% to <13% by the time the disease arrived in the Moray Firth, Scotland [8]. A similar pattern was observed in 2002: mortality declined from 66% in Denmark and Sweden to <1% in Scotland [8].

There were other regional differences in the observed and estimated epidemiological parameters within and between the epidemics [7], [8], [11]. For example, DOE was much longer in Scotland than in many mainland European regions in both epidemics. In most regions, DOEs were shorter in 2002 than 1988 [8] and, in many regions (England, Scotland, the Kattegat, and the Baltic), overall mortality rates were lower [8], [12]. For example, mortality in the Wash (England) was 50% in 1988 but only 22% in 2002 [12].

A number of hypotheses have been put forward to explain these inter- and intra-epidemic differences [7], [13]–[19]. [9] suggested that the within-epidemic differences observed in 1988 were due to differences in either case mortality or contact rate, and [11] concluded that case mortality was the most likely explanation for the regional differences observed within the UK. Differential case mortality could be a result of differences in genetic diversity [7], [16], [17] or population health, which itself might be consequence of pollutant burden [13]–[15].

[8] suggested that the lower levels of mortality observed in some regions in 2002 may have been partly due to the presence of immune survivors from the 1988 epidemic. However, the prevalence of immunity in 2002 was likely to be low in most regions, given the time difference and the level of population growth between the two epidemics [6], [10], [12]. Some authors [18]–[19] have suggested that contact rate may have been affected by seasonal differences in behaviour, because harbour seals spend a greater proportion of time ashore during the breeding season and the annual moult [20]. The reduction in the spread of the disease observed in both epidemics did coincide with the end of the moult period and, in both years, fade-out occurred during the winter months.

The pattern of animal movements, and the spatial network within which these movements take place, are important determinants of the contact process and are an essential component of any spatially-explicit epidemiological model [21]–[26]. Temporally- and spatially-explicit individual-based or cellular automaton models have been used extensively in the modelling of wildlife and livestock diseases (eg. [27], [28]), and provide one way to improve our understanding of the way in which PDV spread. Although [29] questioned whether marine epidemics can be modelled in the same way as terrestrial epidemics because of the greater complexity and openness of the marine environment, a range of marking techniques [22], [30]–[34] have provided data that can be used to estimate the connectivity of local harbour seal populations. As a result, approaches used for terrestrial diseases can be applied to PDV, provided it is only transmitted when infectious individuals are ashore [7], [9], [14]. This assumption appears to be justifiable, because PDV, like other morbilliviruses, is spread by aerosol transmission.

[35] point out that simulation modelling is not an attempt to recreate the world, but rather a tool that can be used to understand how complex systems operate. They suggest that the outputs of simulation can provide the “virtual ecologist” with a “signature” of the set of observations that are likely to be associated with a particular combination of contributing factors. Here, we use this approach to identify the ecological and epidemiological factors that were most likely to be responsible for the differences observed within and between the 1988 and 2002 PDV epidemics.

## Methods

### Model Overview

We developed a stochastic, individual-based, spatially- and temporally-explicit framework to model the spread of PDV through a network of harbour seal haulout sites. We used a real network of over 600 sites identified by [36] during aerial surveys of the island groups of Orkney (59°01′N, 3°06′W) and Shetland (60°23′N, 1°14′W) made in August 2001, when the greatest proportion of animals is assumed to be on land. [36] counted 12,500 seals, which equates to a population of around 18,000 if 60% of the population was on land at the time of the survey [37]. We assumed the sex and age-structure of the harbour seal population in Orkney and Shetland was similar to that of populations in the Kattegat and Skagerrak [38], with a 50∶50 sex ratio and a 36∶64 juvenile∶adult ratio. The 600 sites were clustered into 61 model haulouts, such that no site was further than 15km from the centre of its designated haulout. Each individual seal in the population was randomly allocated to one of these model haulouts.

### Model Parameterisation

**Figure 1:**
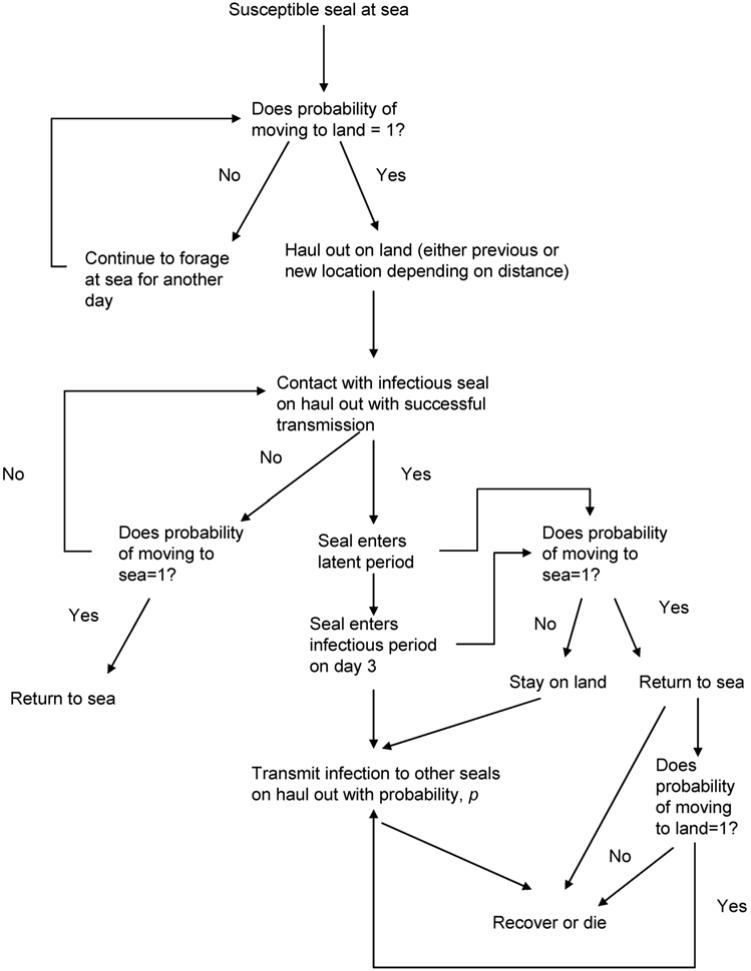
A schematic diagram showing the progression of an individual through the four stages of the SEIR model and the movements between the sea and land.

Following the approach of previous investigations [8], [9], [14], [18], [39], we used a standard SEIR (Susceptible, Exposed, Infective, Resistant) model. The resistant stage within this model includes both those individuals that survive and become immune to the disease (R), and those that die (D) and are thus removed from the population. At the start of each simulation (Julian day 1), all individuals were categorised as being susceptible. On each day of the simulation, each individual was allocated to land or sea depending on its location the previous day. The probability of going to sea increased with the number of days already spent on land, mimicking an increase in the requirement to forage. An equivalent process was used to model the probability of hauling out as the number of days spent at sea increased. When an individual moved from being at sea to being on land, it was allocated to one of the model haulouts using the movement model described below. On a predetermined day within each simulation, one randomly-chosen seal was exposed to the disease (Figure 1). After a latent period of 3 days [18], [40] the infected individual moved into the infectious phase, during which it could transmit the disease to other seals if it was on land (Figure 1). The probability of transmission was modelled using the mass action formula of [23], in which the daily probability of infection was divided by the number of individuals present on the same model haulout as the infectious individual. Infectious animals tend to be lethargic and find it difficult to swim and dive [4], [41], [42]. We therefore assumed that the probability of going to sea was lower during the infectious period. At the end of its infectious period, an individual either died (with a probability determined by the case mortality) or recovered (Figure 1).

The movement of individuals between haulouts followed a model fitted by [43] to data from radio-tagged harbour seals in Orkney and Shetland. This model relates the probability of movements between two haulouts to the distances that a seal would be required to travel between them. It has two components: a continuous dispersal kernel (*L*(*d_ij_*) : **R**_+_ ↦ **R**_+_), which predicts the likelihood of movement between haulouts *i* and *j*; and a normalization *P_ij_* of these likelihoods such that 0< *P_ij_* ≤1 and Σ *P_ij_* = 1. The chosen form of the kernel was:
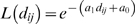
(1)where, *a*_0_ and *a*_1_ were parameters estimated from the data. The chosen form of normalization maintains the proportional relationship between the individual likelihoods, implying that seals base their decisions on where to move on the distances between the haulout they are currently at and those that are available:
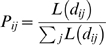
(2)


We set up two factorial design experiments to determine the influence of three epidemiological parameters (contact rate, length of infectious period, and case mortality – Table 1) and three ecological ones (day that infection was introduced into the population, duration of foraging trips, and population size – Table 2). We tested the robustness of the model behaviour to uncertainty by repeating the ecological experiment under different assumptions.

**Table 1:** The 12 treatments modelled in Experiment 1.

Treatment	Contact rate	Case mortality	Length of the infectious period
1	1	0.1	5
2	1	0.1	16
3	1	0.6	5
4	1	0.6	16
5	2	0.1	5
6	2	0.1	16
7	2	0.6	5
8	2	0.6	16
9	3	0.1	5
10	3	0.1	16
11	3	0.6	5
12	3	0.6	16

**Table 2:** The 12 treatments modelled in Experiments 2, 3 and 4.

Treatment	Population size	Day infection introduced	Trip duration
1	10000	10	4
2	10000	10	10
3	10000	100	4
4	10000	100	10
5	10000	200	4
6	10000	200	10
7	20000	10	4
8	20000	10	10
9	20000	100	4
10	20000	100	10
11	20000	200	4
12	20000	200	10

### Experiment 1 - Epidemiological Parameters

The experimental design included three values for contact rate, two values for infectious period and two values for case mortality (Table 1). All values used were within the ranges reported in the literature. We ran 50 replicates of each treatment. The date of introduction was held constant at Julian day 10, and the population size was always 18,000. All individuals spent approximately one-third of their time hauled out on land and had a mean foraging trip duration of 4 days. Each replicate was run until no new individuals became infected and all infectious individuals had either recovered or died. We collected the following data at the end of each simulated day: number of individuals on land, number of infectious individuals and number of dead individuals.

### Experiment 2 - Ecological parameters

The design for this experiment included two values for foraging trip duration (the mean number of days an individual spends at sea before hauling out on land), three values for day of introduction (the Julian day on which the first seal became infected) and two values for population size (Table 2). To ensure that foraging trip duration did not influence contact rate by changing the number of days an animals spends on land, the proportion of time spent at sea was held constant. Thus, under a regime of short foraging trips, animals also spent only short periods of time on land. Changing foraging trip duration therefore had its main effect on connectivity: when foraging trips were short animals made more frequent trips to sea, thus increasing their probability of moving to a new haulout location. In order to assess the affect of day of introduction, the proportion of time hauled out was allowed to vary seasonally according to published values [12], [32], [37]. The proportion of time spent hauled out increased from 0.2 between January and June to 0.35 in July and 0.6 in August. The peak in August was followed by a sharp decline to 0.1 in September. This value was maintained until the following January. A contact rate of 3, a case mortality of 0.3 and an infectious period of 12 days was used throughout this experiment.

### Experiments 3 and 4

A number of other parameters were kept constant throughout Experiments 1 and 2. To test sensitivity to these assumptions, we repeated Experiment 2 with different forms of the mass action function, and different assumptions about the behaviour of infectious animals.

In Experiment 3, we repeated Experiment 2 with a modified mass action function in which the daily probability of infection was divided by the mean number of individuals present on a haulout over the course of the year, rather than the number present each day.

In Experiment 4, we repeated Experiment 2 with identical haulout behaviour for all individuals, regardless of infectious status.

### Model Analysis

We examined five response variables: total number of dead individuals, DOE, PMD, prevalence of immune individuals in the surviving population, and the proportion of replicates where more than 100 individuals died from the disease (detectable epidemics). The first four variables provided us with a signature that could be compared with field observations. We were only interested in those replicates where the disease spread through the population and caused a detectable level of mortality. Including replicates where the disease faded out soon after its introduction would have biased the results by including undetectable levels of mortality.

Each response was modelled as a generalised linear model (GLM). The number of dead individuals and DOE were modelled using the quasipoisson distribution, PMD was modelled using the gamma distribution, prevalence was modelled using the quasibinomial distribution and the proportion of replicates that involved more than 100 deaths was modelled using the binomial (Experiment 1) or the quasibinomial distribution (Experiments 2, 3 and 4). Each model was first fitted without interactions. The output from each model (mean and standard deviation of the response variable) was then plotted against each explanatory variable in combination with the other explanatory variables to determine its biological significance and to identify possible interactions. Because statistical power will be influenced by the number of replicates, we interpreted the probability values from the GLMs with caution and verified the existence of significant relationships using plots. We then incorporated any interaction terms in a forward step-wise manner, and tested the significance of each interaction term using analysis of variance. Plots were again used to further examine the influence and biological significance of any interactions that were statistically significant. Although interactions are identified in all model output tables, they will only be discussed further in cases where the overall signature associated with a particular explanatory variable changed when interactions were included in the model.

### Signature Comparison

Epidemic signatures resulting from the observed number of dead animals, DOE, PMD and prevalence were generated for a selection of locations that were affected by the epidemic in both years from records of the numbers of dead seals and the dates when they were found [8, Harding et al., unpublished data]. Harding et al. (unpublished data) calculated DOE from cumulative death curves [8]. For these cases, we assumed that PMD changed in accordance with DOE.

## Results

### Experiment 1

Contact rate had a significant effect on all five response variables (Table 3). The number of dead individuals, prevalence and the proportion of replicates where a detectable epidemic occurred all increased with increasing contact rate, whereas DOE and PMD decreased with increasing contact rate.

**Table 3:** Results of the Generalised Linear Models fitted to each response variable under the conditions of Experiment 1.

Response variable	Explanatory factors						Significant interactions
	Contact rate		Length of infectious period		Case mortality		
	Significance	Relationship	Significance	Relationship	Significance	Relationship	
Number of dead	*	+ve	NS		*	+ve	Contact*mort* infect
Duration of epidemic	*	−ve	*	+ve	NS		Contact*infect
Peak mortality date	*	−ve	*	+ve	*	+ve	Contact*infect Contact*mort
Prevalence	*	+ve	NS		NS		Contact*infect
Proportion of replicates that spread	*	+ve	*	+ve	*	+ve	

* = significant (P<0.05), NS = not significant (p>0.05), +ve = positive relationship, −ve = negative relationship.

DOE, PMD and the proportion of detectable epidemics all increased with increasing length of the infectious period, but only when contact rate was low and case mortality was high. There was no significant relationship between length of the infectious period and the number of dead individuals or the prevalence of immune individuals in the post-epidemic population.

Number of dead individuals, PMD and proportion of detectable epidemics all increased with increasing case mortality, although PMD only increased when contact rate was low. The increase in the number of dead individuals with increasing mortality was greatest when contact rate was high.

### Experiment 2

Trip duration had a significant effect on four out of the five response variables (Table 4). Number of dead individuals, prevalence and proportion of detectable epidemics all decreased with increasing trip duration and DOE.

**Table 4:** Results of the Generalised Linear Models fitted to each response variable under the conditions of Experiment 2, 3 and 4.

Response variable	Explanatory factors						Significant interactions
	Trip duration		Day of introduction		Population size		
	Significance	Relationship	Significance	Relationship	Significance	Relationship	
**Number of dead**							
Experiment 2	*	−ve	NS		*	+ve	
Experiment 3	*	−ve	NS		*	+ve	Trip* intro Intro*popn
Experiment 4	*	−ve	*	+ve	*	+ve	Trip* intro
**DOE**							
Experiment 2	*	+ve	*	+ve	*	+ve	Intro*popn Trip*intro
Experiment 3	*	+ve	NS		NS		Intro*popn Trip*popn
Experiment 4	*	+ve	*	+ve	*	+ve	Intro*popn
**PMD**							
Experiment 2	NS		*	+ve	NS		
Experiment 3	*	+ve	*	−ve	NS		Intro*popn
Experiment 4	NS		*	+ve	NS		Trip*intro Intro*popn
**Prevalence**							
Experiment 2	*	−ve	NS		NS		
Experiment 3	*	−ve	NS		NS		Trip*intro Intro*popn
Experiment 4	*	−ve	*	+ve	*	+ve	Trip*intro
**Proportion that spread**							
Experiment 2	*	−ve	*	+ve	NS		
Experiment 3	NS		*	+ve	NS		
Experiment 4	NS		NS		*	+ve	Trip*intro

* = significant (P<0.05), NS = not significant (p>0.05) , +ve = positive relationship, −ve = negative relationship.

DOE, PMD and the proportion of detectable epidemics all increased with increasing day of introduction, although the relationship between DOE and day of introduction was dependent on population size. With a small population, the DOE was longest when PDV was introduced late in the year (day 200). With a large population, the DOE was longest when PDV was introduced early in the year (day 100). Population size also affected the number of dead individuals.

### Experiment 3

The main effect of relaxing the mass action assumption was to increase the number of significant interaction terms (Table 4): only the model fitted to the proportion of detectable epidemics had no significant interactions.

The relationship between trip duration and PMD was significant and positive, while the relationship with proportion of detectable epidemics was no longer significant. The relationship between day of introduction and DOE was no longer significant, and the relationship with PMD changed from positive to negative, but only when population size was small. In an interaction with trip duration and population size, day of introduction altered the relationship between trip duration and DOE. The number of dead individuals decreased with increasing trip duration, except when PDV was introduced on Julian day 200, when the number of dead individuals increased with increasing trip duration.

The relationship between population size and DOE was no longer significant. In an interaction with trip duration and day of introduction, population size had an effect on the number of dead individuals. The greatest increase in the number of dead individuals with increasing population size occurred when the day of introduction was 100.

### Experiment 4

Relaxing the assumption relating to the behaviour of infected individuals also increased the number of significant interactions (Table 4). The relationship between trip duration and the proportion of detectable epidemics was no longer significant, whereas the relationships between day of introduction and the number of dead individuals and day of introduction and prevalence became significant and positive. The relationship between day of introduction and the proportion of replicates that spread was no longer significant, although there was a significant interaction between day of introduction and trip duration whereby the proportion of replicates that spread increased between day 100 and 200 when trip duration was short, but there was no increase when trip duration was long.

The relationships between population size and prevalence, and between population size and the proportion of detectable epidemics became significant and positive.

In the model fitted to the DOE, there was no longer a significant interaction between trip duration and day of introduction.

In the model fitted to PMD, there was a significant interaction between length of foraging trip and day of introduction, and a significant interaction between day of introduction and population size. When foraging trip duration was 10 days and population size was 20,000 the largest PMD was when the epidemic was introduced on day 100, whereas under all other treatments the largest PMD was when the epidemic was introduced on day 200.

### Signature Comparison

Table 5 summarises the signatures of the observable parameters for all four experiments. Tables 6 and 7 allow for comparison of these signatures with observations made during the two epidemics (Harding et al., unpublished data). For seven of the regions, the signature was consistent with either a change in contact rate or duration of foraging trips between years (Tables 5 & 6). The signatures for the Onsala, Anholt, Laeso, Wash and Tay regions all indicate that either contact rate was lower or foraging trips were longer in 2002 compared with 1988. For the Wadden Sea NS and NL regions, the signature indicates an increase in contact rate or a decrease in foraging trip duration in 2002 compared with 1988. For all other regions, the signatures suggest that the epidemic started earlier in 2002 than in 1988 in N. Skagerrak, Limfjord, Waddensea DK and the Moray Firth, and later in Halland, Samso and the Baltic.

**Table 5:** Model signatures for an increase in each factor under each experiment.

Factor	Change in Number of Dead	Change in DOE	Change in PMD	Change in Prevalence
**Experiment 1**				
Contact rate	+ve	−ve	−ve	+ve
Length of infectious period	NS	+ve	+ve	NS
Case mortality	+ve	NS	+ve	NS
**Experiment 2**				
Trip duration	−ve	+ve	NS	−ve
Day of introduction	NS	+ve	+ve	NS
Population size	+ve	+ve	NS	NS
**Experiment 3**				
Trip duration	−ve	+ve	+ve	−ve
Day of Introduction	NS	NS	−ve	NS
Population size	+ve	NS	NS	NS
**Experiment 4**				
Trip duration	−ve	+ve	NS	−ve
Day of introduction	+ve	+ve	+ve	+ve
Population size	+ve	+ve	NS	+ve

+ve = positive change, −ve = negative change, NS = no significant change.

**Table 6:** Signatures for the between year changes in epidemic parameters within each region.

Location	Change in Number of Dead	Change in DOE	Change in PMD	Change in Prevalence
N Skagerrak	−ve	−ve	−ve	Unknown
Onsala	−ve	+ve	+ve	Unknown
Halland	+ve	+ve	+ve	Unknown
Anholt	−ve	+ve	+ve	Unknown
Laeso	−ve	+ve	+ve	Unknown
Samso	+ve	+ve	+ve	Unknown
Limfjord	−ve	−ve	−ve	Unknown
Baltic	+ve	+ve	+ve	Unknown
Waddensea DK	−ve	−ve	−ve	Unknown
Wadden Sea NS	+ve	−ve	−ve	Unknown
Wadden Sea NL	+ve	−ve	−ve	Unknown
The Wash	−ve	+ve	+ve	−ve
Tay	−ve	+ve	+ve	−ve
Moray Firth	−ve	−ve	−ve	−ve

+ve = positive change, −ve = negative change, NS = no significant change.

Direction of change is from 1988 to 2002, therefore +ve relates to an increase in a parameter in 2002 relative to 1988. Prevalence is unknown for most regions.

**Table 7:** Signatures for between region differences in epidemic parameters within 2002.

Location	Change in Number of Dead	Change in DOE	Change in PMD	Change in Prevalence
Wadden Sea NL vs Tay	−ve	+ve	+ve	Unknown
Limfjord vs Baltic (option 1)	−ve	+ve	+ve	Unknown
Limfjord vs Baltic (option 2)	NS	+ve	+ve	Unknown
Wash vs Moray Firth	−ve	+ve	+ve	NS

+ve = positive change, −ve = negative change, NS = no significant change.

The direction of change is for the second region relative to the first. Two possible signatures have been included for Limfjord versus the Baltic region because the difference in the number of dead seals relative to population size was marginal.

Within the 2002 epidemic, the observed differences between the Wadden Sea NL and Tay regions are consistent with a lower contact rate or greater foraging trip duration in the Tay (Table 7). Similarly, the differences between Limfjord and the Baltic were also consistent with a greater trip duration or lower contact rate in Limfjord. However, because the difference in mortality between these regions was less than 2%, the results are also consistent with the epidemic arriving later in the Baltic than in Limfjord, or a longer infectious period in the Baltic (Table 7). No model signatures matched the observations from the N. Skagerrak, Limfjord, Waddensea DK and the Moray Firth.

## Discussion

We developed a spatially-explicit, individual-based simulation model of the spread of PDV through local populations of harbour seals that allowed us to assess the role of a number of epidemiological and ecological factors in the 1988 and 2002 European epidemics. This made it possible to incorporate space, time and animal behaviour into a traditional SEIR framework.

Although the model was based on a real spatial network of seal haulouts, we did not try to duplicate the spread of the epidemics through the Orkney/Shetland area, partly because very few dead animals were observed in these regions, even though infected animals were detected there [11]. We used this network as the basis for our simulations because detailed information on the movement of seals between haulouts [33] and an appropriate movement model [43] were available, allowing us to simulate seal movement in a realistic manner.

### Signature comparison

Modelling four different response variables allowed us to create signatures for the expected effect of each explanatory variable. We were then able to use these signatures to identify which hypotheses best explained the observed differences between regions and epidemics. In a number of regions the observed differences indicated that the contact rate in 2002 was lower than in 1988, supporting results from an analysis of antibody prevalence and mortality data from the UK [11], [25].

There are a number of factors that may have altered contact rate between years, such as a change in food availability that resulted in a modification in the way individuals divide their time between foraging at sea and hauling out on land. The continued decline in harbour seal abundance that has been observed in parts of the UK since 2002 [44] lends some support to this explanation.

We were unable to match any of our predicted signatures with the difference observed between two UK regions, the Wash and the Moray Firth, in 2002. There is no evidence that contact rate differed between these regions [11], and harbour seals tagged in the Wash made longer foraging trips than those tagged in the Moray Firth [34]: the opposite of what would be predicted from the signatures.

The increase in the number of dead individuals and the longer DOE that was observed in the Baltic in 2002 suggests that the epidemic reached this region at a later date in this year. This is supported by observations in [8]. The observed differences between the Baltic and Limfjord are consistent with either a later start date or longer infectious period in the Baltic. However, dead animals were discovered in the Baltic 2 months before the first dead animals were found in Limfjord. This suggests that Baltic seals had a longer infectious period, possibly as a result of their different immunological status.

### Testing assumptions

In Experiment 2 we explicitly modelled seasonal changes in seal haulout behaviour, and we therefore expected that the day on which infection was introduced into a population would affect the characteristics of the subsequent epidemic. However, day of infection has no significant effects, possibly because we did not allow density within a haulout to vary. When we did allow density to vary during the year, there was a slight increase in the number of dead individuals but this was not significantly related to the day of introduction.

### Probability of an epidemic occurring

We used our model outputs to investigate how epidemiological and ecological factors affect the probability that a disease outbreak will result in detectable levels of mortality. Short foraging trips coupled with short haulout durations increased the connectivity between haulouts and increased the likelihood that disease would spread between haulouts. Long infectious periods had a similar effect on the spread of the disease. They increased the probability that an individual would spend time on land and/or move between haulout locations during its infectious period Low case mortality not only resulted in smaller numbers of dead animals but it also increased the numbers of immune individuals in the population, thus reducing the effective contact rate and slowing the progress of the epidemic

### Conclusions

The combination of individual-based spatially–explicit models and epidemic signatures provided a useful tool for identifying the epidemiological and ecological factors that may have been responsible for observed differences within and between the two PDV epidemics. This method is likely to be particularly useful in the study of wildlife diseases, but it could also be applied to livestock diseases in which wildlife species act as vectors [27], [28]. This approach is complementary to more commonly used SEIR models [9], [14], [18], [39], and the use of both models in combination may provide further insights into a range of wildlife diseases. PDV is one of the best studied wildlife diseases, and this knowledge enabled us to investigate the unseen factors that may have caused the marked regional differences that were observed within and between epidemics. This study has shown that factors which influence contact rate between individuals have the biggest impact on the spread of the disease, and that animal behaviour and spatial connectivity are likely to be crucial components in models of disease spread.
